# Human cytomegalovirus genomics and transcriptomics through the lens of next-generation sequencing: revision and future challenges

**DOI:** 10.1007/s11262-018-1627-3

**Published:** 2019-01-02

**Authors:** Joan Martí-Carreras, Piet Maes

**Affiliations:** Zoonotic Infectious Diseases Unit, Department of Microbiology and Immunology, Rega Institute, KU Leuven, Herestraat 49, Box 1040, 3000 Leuven, Belgium

**Keywords:** Human cytomegalovirus, Human herpesvirus 5, Genomics, Transcriptomics, Long-read sequencing, Genetic variability

## Abstract

The human cytomegalovirus (HCMV) genome was sequenced by hierarchical shotgun almost 30 years ago. Over these years, low and high passaged strains have been sequenced, improving, albeit still far from complete, the understanding of the coding potential, expression dynamics and diversity of *wild-type* HCMV strains. Next-generation sequencing (NGS) platforms have enabled a huge advancement, facilitating the comparison of differentially passaged strains, challenging diagnostics and research based on a single or reduced gene set genotyping. In addition, it allowed to link genetic features to different viral phenotypes as for example, correlating large genomic re-arrangements to viral attenuation or different mutations to antiviral resistance and cell tropism. NGS platforms provided the first high-resolution experiments to HCMV dynamics, allowing the study of intra-host viral population structures and the description of rare transcriptional events. Long-read sequencing has recently become available, helping to identify new genomic re-arrangements, partially accounting for the genetic variability displayed in clinical isolates, as well as, in changing the understanding of the HCMV transcriptome. Better knowledge of the transcriptome resulted in a vast number of new splicing events and alternative transcripts, although most of them still need additional validation. This review summarizes the sequencing efforts reached so far, discussing its approaches and providing a revision and new nuances on HCMV sequence variability in the sequencing field.

## Introduction

In 1881, Hugo Ribbert found the first evidence of cytomegalia and body inclusions in kidney and paratiroid gland cells [1]. Nevertheless, it was only in 1904, and in parallel with Jesionek and Kiolemenoglu, that the evidence was properly reported [1, 2]. Years later, between 1956 and 1957 Smith, Rowe and Weller collaborated in the isolation of the virus, known thereafter as “cytomegalovirus” [3–5]. In 1984, 28 years after its first isolation, the first sequence of human cytomegalovirus or HCMV (strain AD169) was published [6], and only 6 years after, in 1990, the first draft of an annotated HCMV genome was published [7], at that time the biggest contiguous genome sequenced (GenBank accession number BK000394.5, additional information in Table 1). Since 1990 and until the submission of this original work, 305 full-length *distinct* complete HCMV genomes have been published, including low and high passaged strains, lab-attenuated strains, or artificial genomes (NIAID Virus Pathogen Database and Analysis Resource, ViPR) [8].

**Table 1:** Full-length HCMV genomes from clinical isolates

Genbank	Strain	Sample origin	Passage history	Length	Origin	Isolation year	Sequencing platform	Enrichment	References
AC146851	Towne varS-BAC	Urine	> 125	229,483	USA	1970	Sanger	Unenriched	[9]
AC146904	PH-BAC	Bone marrow	< 12	229,700	USA	1984	Sanger	Unenriched	[9]
AC146905	Toledo-BAC	Urine	Highly-passaged	226,889	USA	1984	Sanger	Unenriched	[9]
AC146906	TR-BAC	Ocular isolate	Highly-passaged	234,881	USA	1994	Sanger	Unenriched	[9]
AC146907	FIX (VR1814)-BAC	Cervical secretion	Highly-passaged	229,209	Italy	1996	Sanger	Unenriched	[9]
AC146999	AD169 varATCC-BAC	Adenoids	Highly-passaged	233,739	USA	1956	Sanger	Unenriched	[9]
AY315197	Towne varS-BAC	Urine	Highly-passaged	222,047	USA	1970	Sanger	Unenriched	[10]
AY446894	Merlin	Urine	3	235,646	UK	1999	Sanger	Unenriched	[11]
BK000394	AD169 varUK	Adenoids	Highly-passaged	230,290	USA	1956	Sanger	Unenriched	[7]
EF999921	TB40-BAC4	Throat wash	27	229,050	Germany	1999	Sanger	Unenriched	[12]
FJ527563	AD169 varUC	Adenoids	> 50	231,781	USA	1956	Illumina	Unenriched	[13]
FJ616285	Towne varL	Urine	Highly-passaged	235,147	USA	1970	Illumina	Unenriched	[14]
GQ221973	HAN13	Bronchoalveolar lavage	3	236,219	Germany	2007	Sanger, Illumina	Unenriched	[15]
GQ221974	3157	Urine	3	235,154	UK	2001	Sanger	Unenriched	[15]
GQ221975	JP	Prostate tissue	Unpassaged	236,375	UK	2001	Sanger	Unenriched	[15]
GQ396662	HAN38	Bronchoalveolar lavage	2	236,112	Germany	2007	Illumina	Unenriched	[15]
GQ396663	HAN20	Bronchoalveolar lavage	2	235,728	UK	2007	Illumina	Unenriched	[15]
GQ466044	3301	Urine	Unpassaged	235,703	UK	2001	Illumina	Unenriched	[15]
GU179288	U8	Urine	> 50	235,709	Italy	2003	Sanger	Unenriched	[16]
GU179289	VR1814	Cervical secretion	> 154	235,233	Italy	1996	Sanger	Unenriched	[16]
GU179290	U11	Urine	> 50	234,732	UK	2003	Sanger	Unenriched	[16]
GU179291	AF1	Amniotic fluid	> 50	235,937	Italy	2003	Sanger	Unenriched	[16]
GU937742	Toledo	Urine	Highly-passaged	235,398	USA	1984	Sanger	Unenriched	[11]
HQ380895	JHC	Blood	4	235,476	South Korea	2003	Sanger, 454	Unenriched	[17]
JN379814	U01	Urine	Unpassaged	232,216	USA	2008	Amplicon Illumina	Unenriched	[18]
JN379815	U04	Urine	Unpassaged	233,910	USA	2009	Amplicon Illumina	Unenriched	[18]
JN379816	U33	Urine	Unpassaged	232,889	USA	2009	Amplicon Illumina	Unenriched	[18]
JX512197	6397	Urine	3	235,870	UK	2001	Sanger	Unenriched	[14]
JX512198	Davis	Liver biopsy	Highly-passaged	229,768	USA	1957	Sanger	Unenriched	[14]
JX512199	HAN1	Bronchoalveolar lavage	< 5	235,006	Germany	2007	Unknown	Unenriched	Davison unpublished
JX512200	HAN2	Bronchoalveolar lavage	3	232,940	Germany	2007	Unknown	Unenriched	Davison unpublished
JX512201	HAN3	Bronchoalveolar lavage	3	235,703	Germany	2007	Unknown	Unenriched	Davison unpublished
JX512202	HAN8	Bronchoalveolar lavage	3	234,951	Germany	2007	Unknown	Unenriched	Davison unpublished
JX512203	HAN12	Bronchoalveolar lavage	3	236,006	Germany	2007	Unknown	Unenriched	Davison unpublished
JX512204	HAN16	Urine	2	235,112	Germany	2007	Unknown	Unenriched	Davison unpublished
JX512205	HAN19	Bronchoalveolar lavage	2	235,810	Germany	2007	Unknown	Unenriched	Davison unpublished
JX512206	HAN22	Bronchoalveolar lavage	2	236,379	Germany	2007	Unknown	Unenriched	Davison unpublished
JX512207	HAN28	Bronchoalveolar lavage	3	236,017	Germany	2007	Unknown	Unenriched	Davison unpublished
JX512208	HAN31	Bronchoalveolar lavage	2	235,720	Germany	2007	Unknown	Unenriched	Davison unpublished
KC519319	BE/9/2010	Urine	2	235,631	Belgium	2010	Sanger, 454, Illumina	Multiple displacement amplification	[19]
KC519320	BE/10/2010	Urine	2	235,215	Belgium	2010	454, Illumina	Multiple displacement amplification	[19]
KC519321	BE/11/2010	Urine	2	235,061	Belgium	2010	Sanger, 454, Illumina	Multiple displacement amplification	[19]
KC519322	BE/21/2010	Urine	Unpassaged	235,722	Belgium	2010	Sanger, 454, Illumina	Multiple displacement amplification	[19]
KC519323	BE/27/2010	Urine	4	234,810	Belgium	2010	454, Illumina	Multiple displacement amplification	[19]
KF021605	TR	Vitreous	Highly-passaged	235,681	USA	1996	Sanger	Unenriched	[20]
KF297339	TB40/E clone Lisa	Throat wash	4	237,683	Germany	1999	Sanger	Unenriched	[21]
KJ361946	2CEN2	Bronchoalveolar lavage	1	235,360	Germany	2009	Unknown	Unenriched	Wilkie unpublished
KJ361947	2CEN5	Bronchoalveolar lavage	1	235,567	Germany	2009	Unknown	Unenriched	Wilkie unpublished
KJ361948	2CEN15	Bronchoalveolar lavage	1	234,949	Germany	Unknown	Unknown	Unenriched	Wilkie unpublished
KJ361949	2CEN30	Bronchoalveolar lavage	1	236,168	Germany	Unknown	Unknown	Unenriched	Wilkie unpublished
KJ361950	HAN11	Bronchoalveolar lavage	3	235,276	Germany	2007	Unknown	Unenriched	Davison unpublished
KJ361951	HAN21	Bronchoalveolar lavage	3	235,834	Germany	2006	Unknown	Unenriched	Davison unpublished
KJ361952	HAN27	Bronchoalveolar lavage	2	235,861	Germany	2007	Unknown	Unenriched	Davison unpublished
KJ361953	HAN30	Bronchoalveolar lavage	2	235,483	Germany	2006	Unknown	Unenriched	Davison unpublished
KJ361954	HAN32	Bronchoalveolar lavage	2	235,458	Germany	2007	Unknown	Unenriched	Davison unpublished
KJ361955	HAN33	Bronchoalveolar lavage	3	235,512	Germany	2007	Unknown	Unenriched	Davison unpublished
KJ361956	HAN36	Bronchoalveolar lavage	2	234,844	Germany	2007	Unknown	Unenriched	Davison unpublished
KJ361957	HAN39	Bronchoalveolar lavage	1	235,056	Germany	2007	Unknown	Unenriched	Davison unpublished
KJ361958	HAN40	Bronchoalveolar lavage	2	235,763	Germany	2007	Unknown	Unenriched	Davison unpublished
KJ361959	PAV1	Amniotic fluid	Unpassaged	235,815	Italy	2005	Unknown	Unenriched	Wilkie unpublished
KJ361960	PAV4	Amniotic fluid	Unpassaged	235,272	Italy	2006	Unknown	Unenriched	Wilkie unpublished
KJ361961	PAV5	Amniotic fluid	Unpassaged	235,485	Italy	2006	Unknown	Unenriched	Wilkie unpublished
KJ361962	PAV6	Amniotic fluid	Unpassaged	235,432	Italy	2007	Unknown	Unenriched	Wilkie unpublished
KJ361963	PAV7	Amniotic fluid	Unpassaged	235,142	Italy	2007	Unknown	Unenriched	Wilkie unpublished
KJ361964	PAV8	Amniotic fluid	Unpassaged	235,432	Italy	2007	Unknown	Unenriched	Wilkie unpublished
KJ361965	PAV11	Amniotic fluid	Unpassaged	236,310	Italy	2007	Unknown	Unenriched	Wilkie unpublished
KJ361966	PAV12	Amniotic fluid	Unpassaged	235,616	Italy	2007	Unknown	Unenriched	Wilkie unpublished
KJ361967	PAV23	Amniotic fluid	Unpassaged	235,700	Italy	2012	Unknown	Unenriched	Wilkie unpublished
KJ361968	PAV24	Amniotic fluid	Unpassaged	235,361	Italy	2012	Unknown	Unenriched	Wilkie unpublished
KJ361969	PAV25	Amniotic fluid	Unpassaged	235,902	Italy	2013	Unknown	Unenriched	Wilkie unpublished
KJ361970	PAV26	Amniotic fluid	Unpassaged	236,180	Italy	2013	Unknown	Unenriched	Wilkie unpublished
KJ361971	UKNEQAS1	Urine	2	235,190	UK	2012	Unknown	Target enrichment	Wilkie unpublished
KJ426589	HAN	Clinical isolate	Unknown	236,144	China	2007	Illumina	Unenriched	Ma unpublished
KJ872539	PAV16	Amniotic fluid	Unpassaged	236,240	Italy	2009	Illumina	Target enrichment	Wilkie unpublished
KJ872540	PAV18	Amniotic fluid	Unpassaged	234,739	Italy	2009	Illumina	Target enrichment	Wilkie unpublished
KJ872541	PAV20	Amniotic fluid	Unpassaged	236,293	Italy	2013	Illumina	Target enrichment	Wilkie unpublished
KJ872542	PAV21	Amniotic fluid	Unpassaged	235,901	Italy	2013	Illumina	Target enrichment	Wilkie unpublished
KP745633	BE/45/2011	Nasopharyngeal aspirate	1	235,352	Belgium	2011	Illumina	Multiple displacement amplification	[22]
KP745634	BE/32/2010	Amniotic fluid	1	235,205	Belgium	2010	Illumina	Multiple displacement amplification	[22]
KP745635	BE/5/2012	Urine	2	235,184	Belgium	2012	Illumina	Multiple displacement amplification	[22]
KP745636	BE/7/2011	Urine	2	237,117	Belgium	2011	454, Illumina	Multiple displacement amplification	[22]
KP745637	BE/9/2011	Urine	2	235,865	Belgium	2011	454, Illumina	Multiple displacement amplification	[22]
KP745638	BE/15/2010	Urine	3	235,514	Belgium	2010	454, Illumina	Multiple displacement amplification	[22]
KP745639	BE/10/2011	Urine	2	235,054	Belgium	2011	454, Illumina	Multiple displacement amplification	[22]
KP745640	BE/22/2010	Urine	4	235,632	Belgium	2010	454, Illumina	Multiple displacement amplification	[22]
KP745641	BE/31/2011	Urine	4	235,844	Belgium	2011	Illumina	Multiple displacement amplification	[22]
KP745642	CZ/1/2012	Urine	2	235,030	Czech Republic	2012	Illumina	Multiple displacement amplification	[22]
KP745643	CZ/2/2012	Urine	2	235,226	Czech Republic	2012	Illumina	Multiple displacement amplification	[22]
KP745644	BE/31/2010	Urine	4	236,028	Belgium	2010	454, Illumina	Multiple displacement amplification	[22]
KP745645	BE/13/2010	Urine	3	236,032	Belgium	2010	454, Illumina	Multiple displacement amplification	[22]
KP745646	BE/8/2012	Urine	3	235,889	Belgium	2012	Illumina	Multiple displacement amplification	[22]
KP745647	BE/18/2010	Urine	5	235,871	Belgium	2010	454, Illumina	Multiple displacement amplification	[22]
KP745648	BE/8/2011	Urine	2	235,111	Belgium	2011	454, Illumina	Multiple displacement amplification	[22]
KP745649	BE/10/2012	Urine	2	234,754	Belgium	2012	Illumina	Multiple displacement amplification	[22]
KP745650	BE/1/2011	Urine	3	235,833	Belgium	2011	454, Illumina	Multiple displacement amplification	[22]
KP745651	BE/9/2012	Urine	2	235,836	Belgium	2012	Illumina	Multiple displacement amplification	[22]
KP745652	BE/2/2011	Urine	4	235,810	Belgium	2011	454, Illumina	Multiple displacement amplification	[22]
KP745653	BE/22/2011	Urine	2	235,612	Belgium	2011	Illumina	Multiple displacement amplification	[22]
KP745654	BE/19/2011	Urine	2	235,446	Belgium	2011	Illumina	Multiple displacement amplification	[22]
KP745655	BE/3/2010	Urine	2	236,597	Belgium	2010	454, Illumina	Multiple displacement amplification	[22]
KP745656	BE/2/2013	Urine	3	235,156	Belgium	2013	Illumina	Multiple displacement amplification	[22]
KP745657	BE/13/2011	Urine	2	235,713	Belgium	2011	454, Illumina	Multiple displacement amplification	[22]
KP745658	BE/14/2012	Urine	1	234,931	Belgium	2012	Illumina	Multiple displacement amplification	[22]
KP745659	BE/3/2011	Urine	4	235,726	Belgium	2011	454, Illumina	Multiple displacement amplification	[22]
KP745660	BE/6/2011	Urine	2	235,101	Belgium	2011	454, Illumina	Multiple displacement amplification	[22]
KP745661	BE/33/2010	Nasopharyngeal aspirate	1	235,605	Belgium	2010	Illumina	Multiple displacement amplification	[22]
KP745662	BE/20/2010	Urine	4	235,516	Belgium	2010	454, Illumina	Multiple displacement amplification	[22]
KP745663	BE/5/2010	Urine	2	236,345	Belgium	2010	454, Illumina	Multiple displacement amplification	[22]
KP745664	CZ/2/2013	Blood	2	235,191	Czech Republic	2013	Illumina	Multiple displacement amplification	[22]
KP745665	BE/16/2012	Urine	1	235,910	Belgium	2012	Illumina	Multiple displacement amplification	[22]
KP745666	BE/7/2012	Urine	3	236,053	Belgium	2012	Illumina	Multiple displacement amplification	[22]
KP745667	BE/5/2011	Urine	7	235,621	Belgium	2011	454, Illumina	Multiple displacement amplification	[22]
KP745668	BE/18/2011	Urine	2	235,416	Belgium	2011	454, Illumina	Multiple displacement amplification	[22]
KP745669	BE/28/2011	Nasopharyngeal swab	2	235,732	Belgium	2011	Illumina	Multiple displacement amplification	[22]
KP745670	BE/30/2011	Urine	2	235,350	Belgium	2011	Illumina	Multiple displacement amplification	[22]
KP745671	BE/14/2011	Urine	9	235,498	Belgium	2011	Illumina	Multiple displacement amplification	[22]
KP745672	BE/29/2011	Urine	2	236,364	Belgium	2011	Illumina	Multiple displacement amplification	[22]
KP745673	BE/42/2011	Nasopharyngeal aspirate	1	235,462	Belgium	2011	Illumina	Multiple displacement amplification	[22]
KP745674	BE/33/2011	Urine	2	235,276	Belgium	2011	Illumina	Multiple displacement amplification	[22]
KP745675	BE/23/2011	Nasopharyngeal swab	2	235,425	Belgium	2011	Illumina	Multiple displacement amplification	[22]
KP745676	BE/28/2010	Urine	4	235,974	Belgium	2010	454, Illumina	Multiple displacement amplification	[22]
KP745677	BE/1/2010	Urine	2	235,705	Belgium	2010	Illumina	Multiple displacement amplification	[22]
KP745678	BE/25/2010	Urine	2	235,904	Belgium	2010	454, Illumina	Multiple displacement amplification	[22]
KP745679	BE/24/2010	Urine	2	235,744	Belgium	2010	Illumina	Multiple displacement amplification	[22]
KP745680	BE/11/2012	Urine	2	235,893	Belgium	2012	Illumina	Multiple displacement amplification	[22]
KP745681	BE/43/2011	Nasopharyngeal aspirate	1	235,100	Belgium	2011	Illumina	Multiple displacement amplification	[22]
KP745682	BE/46/2011	Nasopharyngeal aspirate	1	236,239	Belgium	2011	Illumina	Multiple displacement amplification	[22]
KP745683	BE/12/2011	Urine	2	235,258	Belgium	2011	454, Illumina	Multiple displacement amplification	[22]
KP745684	BE/11/2011	Urine	4	234,806	Belgium	2011	454, Illumina	Multiple displacement amplification	[22]
KP745685	CZ/3/2012	Urine	2	234,598	Czech Republic	2012	Illumina	Multiple displacement amplification	[22]
KP745686	BE/39/2011	Nasopharyngeal aspirate	1	235,982	Belgium	2011	Illumina	Multiple displacement amplification	[22]
KP745687	BE/36/2011	Urine	2	234,373	Belgium	2011	Illumina	Multiple displacement amplification	[22]
KP745688	BE/12/2012	Nasopharyngeal swab	2	235,362	Belgium	2012	Illumina	Multiple displacement amplification	[22]
KP745689	BE/17/2011	Urine	2	235,827	Belgium	2011	454, Illumina	Multiple displacement amplification	[22]
KP745690	BE/34/2011	Urine	2	235,290	Belgium	2011	Illumina	Multiple displacement amplification	[22]
KP745691	CZ/1/2013	Blood	2	235,139	Czech Republic	2013	Illumina	Multiple displacement amplification	[22]
KP745692	BE/3/2012	Urine	2	236,051	Belgium	2012	Illumina	Multiple displacement amplification	[22]
KP745693	BE/15/2012	Urine	1	235,508	Belgium	2012	Illumina	Multiple displacement amplification	[22]
KP745694	BE/12/2010	Urine	8	235,195	Belgium	2010	454, Illumina	Multiple displacement amplification	[22]
KP745695	BE/6/2012	Urine	5	235,164	Belgium	2012	Illumina	Multiple displacement amplification	[22]
KP745696	BE/27/2011	Urine	5	235,392	Belgium	2011	Illumina	Multiple displacement amplification	[22]
KP745697	BE/23/2010	Urine	4	236,066	Belgium	2010	454, Illumina	Multiple displacement amplification	[22]
KP745698	BE/20/2011	Urine	2	235,272	Belgium	2011	454, Illumina	Multiple displacement amplification	[22]
KP745699	BE/1/2012	Urine	2	235,150	Belgium	2012	Illumina	Multiple displacement amplification	[22]
KP745700	BE/4/2011	Urine	2	235,808	Belgium	2011	454, Illumina	Multiple displacement amplification	[22]
KP745701	BE/6/2010	Urine	2	235,329	Belgium	2010	454, Illumina	Multiple displacement amplification	[22]
KP745702	BE/21/2011	Urine	5	235,849	Belgium	2011	Illumina	Multiple displacement amplification	[22]
KP745703	BE/26/2011	Urine	2	234,902	Belgium	2011	454, Illumina	Multiple displacement amplification	[22]
KP745704	BE/32/2011	Urine	2	235,633	Belgium	2011	Illumina	Multiple displacement amplification	[22]
KP745705	BE/38/2011	Nasopharyngeal swab	2	235,775	Belgium	2011	Illumina	Multiple Displacement Amplification	[22]
KP745706	BE/41/2011	Bronchoalveolar lavage	1	235,332	Belgium	2011	Illumina	Multiple displacement amplification	[22]
KP745707	BE/13/2012	Urine	2	235,015	Belgium	2012	Illumina	Multiple displacement amplification	[22]
KP745708	BE/8/2010	Urine	1	235,964	Belgium	2010	Illumina	Multiple displacement amplification	[22]
KP745709	BE/48/2011	Nasopharyngeal aspirate	1	235,747	Belgium	2011	Illumina	Multiple displacement amplification	[22]
KP745710	BE/2/2012	Urine	2	236,100	Belgium	2012	Illumina	Multiple displacement amplification	[22]
KP745711	BE/24/2011	Urine	2	235,745	Belgium	2011	454, Illumina	Multiple displacement amplification	[22]
KP745712	BE/19/2010	Urine	5	235,365	Belgium	2010	454, Illumina	Multiple displacement amplification	[22]
KP745713	BE/35/2011	Urine	2	235,941	Belgium	2011	Illumina	Multiple displacement amplification	[22]
KP745714	BE/29/2010	Urine	7	234,922	Belgium	2010	454, Illumina	Multiple displacement amplification	[22]
KP745715	BE/44/2011	Nasopharyngeal aspirate	1	235,301	Belgium	2011	Illumina	Multiple displacement amplification	[22]
KP745716	BE/16/2010	Nasopharyngeal swab	5	235,366	Belgium	2010	454, Illumina	Multiple displacement amplification	[22]
KP745717	BE/2/2010	Nasopharyngeal swab	2	235,138	Belgium	2010	454, Illumina	Multiple displacement amplification	[22]
KP745718	CZ/1/2011	Urine	2	234,758	Czech Republic	2011	Illumina	Multiple displacement amplification	[22]
KP745719	BE/26/2010	Urine	2	235,908	Belgium	2010	454, Illumina	Multiple displacement amplification	[22]
KP745720	BE/15/2011	Urine	5	235,905	Belgium	2011	454, Illumina	Multiple displacement amplification	[22]
KP745721	BE/14/2010	Nasopharyngeal swab	2	234,537	Belgium	2010	454, Illumina	Multiple displacement amplification	[22]
KP745722	BE/40/2011	Nasopharyngeal aspirate	1	235,716	Belgium	2011	Illumina	Multiple displacement amplification	[22]
KP745723	BE/37/2011	Nasopharyngeal swabs	5	234,858	Belgium	2011	Illumina	Multiple displacement amplification	[22]
KP745724	BE/4/2012	Urine	2	234,950	Belgium	2012	Illumina	Multiple displacement amplification	[22]
KP745725	BE/49/2011	Nasopharyngeal aspirate	1	235,317	Belgium	2011	Illumina	Multiple displacement amplification	[22]
KP745726	BE/30/2010	Urine	2	235,642	Belgium	2010	454, Illumina	Multiple displacement amplification	[22]
KP745727	BE/17/2010	Urine	4	235,836	Belgium	2010	454, Illumina	Multiple displacement amplification	[22]
KP745728	BE/4/2010	Urine	2	236,428	Belgium	2010	Illumina	Multiple displacement amplification	[22]
KR534196	JER847	Urine	4	235,713	Israel	2009	Unknown	Unenriched	Wilkie unpublished
KR534197	JER851	Urine	4	235,435	Israel	2009	Unknown	Unenriched	Wilkie unpublished
KR534198	JER893	Bronchoalveolar lavage	3	235,790	Israel	2009	Unknown	Unenriched	Wilkie unpublished
KR534199	JER1070	Amniotic fluid	Unpassaged	235,492	Israel	2010	Unknown	Unenriched	Wilkie unpublished
KR534200	JER1289	kidney biopsy	3	235,841	Israel	2002	Unknown	Unenriched	Wilkie unpublished
KR534201	JER2002	Amniotic fluid	Unpassaged	235,339	Israel	2011	Unknown	Unenriched	Wilkie unpublished
KR534202	JER2282	Amniotic fluid	Unpassaged	234,549	Israel	2012	Unknown	Unenriched	Wilkie unpublished
KR534203	JER3230	Amniotic fluid	Unpassaged	235,857	Israel	2011	Unknown	Unenriched	Wilkie unpublished
KR534204	JER3855	Amniotic fluid	Unpassaged	234,804	Israel	2005	Unknown	Unenriched	Wilkie unpublished
KR534205	JER4035	Amniotic fluid	Unpassaged	235,314	Israel	2009	Unknown	Unenriched	Wilkie unpublished
KR534206	JER4041	Amniotic fluid	Unpassaged	234,917	Israel	2005	Unknown	Unenriched	Wilkie unpublished
KR534207	JER4053	Amniotic fluid	Unpassaged	235,126	Israel	2009	Unknown	Unenriched	Wilkie unpublished
KR534208	JER4559	Amniotic fluid	Unpassaged	235,673	Israel	2009	Unknown	Unenriched	Wilkie unpublished
KR534209	JER4755	Amniotic fluid	Unpassaged	235,266	Israel	2012	Unknown	Unenriched	Wilkie unpublished
KR534210	JER5268	Amniotic fluid	Unpassaged	235,445	Israel	2012	Unknown	Unenriched	Wilkie unpublished
KR534211	JER5409	Amniotic fluid	Unpassaged	235,943	Israel	2012	Unknown	Unenriched	Wilkie unpublished
KR534212	JER5550	Amniotic fluid	Unpassaged	235,160	Israel	2012	Unknown	Unenriched	Wilkie unpublished
KR534213	JER5695	Amniotic fluid	Unpassaged	235,797	Israel	2012	Unknown	Unenriched	Wilkie unpublished
KT634296	UKNEQAS2	Amniotic fluid	Unpassaged	234,873	Australia	2013	Illumina	Target enrichment	Wilkie unpublished
KT726945	NL/Rot6/Nasal/2012	Nasal rinse	1	234,696	The Netherlands	2012	Illumina	Target enrichment	[23]
KT726947	UK/Lon1/Blood/2013	Blood	Unpassaged	235,143	UK	2013	Illumina	Target enrichment	[23]
KT726949	UK/Lon6/Urine/2011	Urine	Unpassaged	235,199	UK	2011	Illumina	Target enrichment	[23]
KT726950	UK/Lon7/Urine/2011	Urine	Unpassaged	235,743	UK	2011	Illumina	Target enrichment	[23]
KT726951	UK/Lon8/Urine/2012	Urine	Unpassaged	235,801	UK	2012	Illumina	Target enrichment	[23]
KT959235	DB	Cervical swab	3	235,512	France	2009	Illumina	Target enrichment	[24]
KU550087	NAN1LA	Amniotic fluid	Unpassaged	235,062	France	2011	Illumina	Target enrichment	Wilkie unpublished
KU550088	NAN2LA	Amniotic fluid	Unpassaged	234,396	France	2013	Illumina	Target enrichment	Wilkie unpublished
KU550089	NAN4LA	Amniotic fluid	Unpassaged	237,120	France	2013	Illumina	Target enrichment	Wilkie unpublished
KU550090	NANU	Urine	Unpassaged	235,634	France	2013	Illumina	Target enrichment	Wilkie unpublished
KX544831	NR	Blood	BAC-cloned	235,133	USA	2016	454	Unenriched	[25]
KX544832	SUB_24	Urine	Highly-passaged	235,534	USA	2016	454	Unenriched	[25]
KX544833	VR3908	Urine	Highly-passaged	234,711	USA	2016	454	Unenriched	[25]
KX544834	SUB_22	Urine	Highly-passaged	233,965	USA	2016	454	Unenriched	[25]
KX544835	VR5022	Blood	Highly-passaged	234,640	USA	2016	454	Unenriched	[25]
KX544836	VR5201	Blood	Highly-passaged	234,660	USA	2016	454	Unenriched	[25]
KX544837	VR5235	Blood	Highly-passaged	235,666	USA	2016	454	Unenriched	[25]
KX544838	VR7863	Urine	Highly-passaged	234,769	USA	2016	454	Unenriched	[25]
KX544839	TB40-E_UNC	Throat swab	BAC-cloned	228,992	USA	2016	454	Unenriched	[25]
KX544840	UXCA_Merck_UNC	Urine	BAC-cloned	223,782	USA	2016	454	Unenriched	[25]
KX544841	VHL-E_Merck_UNC	Duodenal biopsy	NA	222,309	USA	2016	454	Unenriched	[25]
KY002201	Toledo variant	Urine	Highly-passaged	235,681	USA	1984	Illumina	Unenriched	Suárez unpublished
KY123649	HANChild4	Bronchoalveolar secretion	Unpassaged	235,275	Germany	2012	Illumina	Target enrichment	[26]
KY123650	HANRTR2	Blood	Unpassaged	235,472	Germany	2012	Illumina	Target enrichment	[26]
KY123651	HANRTR4	Plasma	Unpassaged	235,329	Germany	2015	Illumina	Target enrichment	[26]
KY123652	HANRTR5	Biopsy	Unpassaged	235,233	Germany	2015	Illumina	Target enrichment	[26]
KY123653	HANSCTR4	Blood	Unpassaged	235,510	Germany	2011	Illumina	Target enrichment	[26]
KY490061	PAV31	Plasma	Unpassaged	235,221	Italy	Unknown	Illumina	Unenriched	Suárez unpublished
KY490062	PAV32	Plasma	Unpassaged	234,316	Italy	Unknown	Illumina	Target enrichment	Suárez unpublished
KY490063	PRA1	Urine	Unpassaged	235,826	Czech Republic	2006	Illumina	Target enrichment	Suárez unpublished
KY490064	PRA2	Urine	Unpassaged	234,791	Czech Republic	2009	Illumina	Target enrichment	Suárez unpublished
KY490065	PRA3	Urine	Unpassaged	235,442	Czech Republic	2009	Illumina	Target enrichment	Suárez unpublished
KY490066	PRA4	Urine	Unpassaged	235,513	Czech Republic	2009	Illumina	Target enrichment	Suárez unpublished
KY490067	PRA5	Urine	Unpassaged	234,989	Czech Republic	2009	Illumina	Target enrichment	Suárez unpublished
KY490068	PRA6	Amniotic fluid	Unpassaged	235,717	Czech Republic	2015	Illumina	Target enrichment	Suárez unpublished
KY490069	PRA7	Urine	Unpassaged	236,373	Czech Republic	2010	Illumina	Target enrichment	Suárez unpublished
KY490070	PRA8	Urine	Unpassaged	234,832	Czech Republic	2012	Illumina	Target enrichment	Suárez unpublished
KY490071	HANChild1	Urine	Unpassaged	235,397	Germany	2013	Illumina	Target enrichment	Suárez unpublished
KY490072	HANChild2&3	Urine	Unpassaged	235,913	Germany	2013	Illumina	Target enrichment	Suárez unpublished
KY490073	HANTR1A	Blood	Unpassaged	235,221	Germany	2012	Illumina	Target enrichment	Suárez unpublished
KY490074	HANTR1B	Blood	Unpassaged	235,385	Germany	2013	Illumina	Target enrichment	Suárez unpublished
KY490075	HANTR6	Vitreous humor	Unpassaged	235,930	Germany	2014	Illumina	Target enrichment	Suárez unpublished
KY490076	HANTR8	Blood	Unpassaged	235,791	Germany	2013	Illumina	Target enrichment	Suárez unpublished
KY490077	HANTR9	Kidney biopsy	Unpassaged	235,175	Germany	2011	Illumina	Target enrichment	Suárez unpublished
KY490078	HANTR10	Blood	Unpassaged	234,360	Germany	2010	Illumina	Target enrichment	Suárez unpublished
KY490079	HANSCTR1A	Blood	Unpassaged	235,579	Germany	2014	Illumina	Target enrichment	Suárez unpublished
KY490080	HANSCTR1B	Stem cell biopsy	Unpassaged	235,688	Germany	2014	Illumina	Target enrichment	Suárez unpublished
KY490081	HANSCTR2	Blood	Unpassaged	235,843	Germany	2015	Illumina	Target enrichment	Suárez unpublished
KY490082	HANSCTR8	Blood	Unpassaged	235,058	Germany	2014	Illumina	Target enrichment	Suárez unpublished
KY490083	HANSCTR9	Blood	Unpassaged	235,153	Germany	2016	Illumina	Target enrichment	Suárez unpublished
KY490084	HANSCTR10	Bronchoalveolar lavage	Unpassaged	235,018	Germany	2013	Illumina	Target enrichment	Suárez unpublished
KY490085	HANSCTR11A	Blood	Unpassaged	235,632	Germany	2010	Illumina	Target enrichment	Suárez unpublished
KY490086	HANSCTR11B	Blood	Unpassaged	234,962	Germany	2010	Illumina	Target enrichment	Suárez unpublished
KY490087	HANSCTR12	Blood	Unpassaged	235,848	Germany	2010	Illumina	Target enrichment	Suárez unpublished
KY490088	HANSCTR13	Blood	Unpassaged	235,403	Germany	2011	Illumina	Target enrichment	Suárez unpublished
LT907985	Towne	Urine	> 125	232,608	USA	1970	PacBio	Unenriched	[27, 28]
MF084223	LON1	Urine	1	235,168	UK	2016	Illumina	Target enrichment	Suárez unpublished
MF084224	HER1	Urine	1	235,079	Greece	2016	Illumina	Target enrichment	Suárez unpublished
X17403	AD169	Adenoids	> 50	229,354	USA	1956	Sanger	Unenriched	[7]

List of all available HCMV genomes derived from clinical isolates extracted from NIAID Virus Pathogen Database and Analysis Resource (ViPR, June 2018) (artificially created mutants have been excluded) [8]. Different fields describe relevant genome information: GenBank accession number, clinical origin of the sample, passage history, genome length, country of isolation, year of isolation, sequencing method to obtain the genome and the enrichment method that was used, if applicable

Human herpesvirus 5 (HHV-5) or HCMV, a member of the family *Herpesviridae* subfamily *Betaherpesvirinae*, is a human-infecting ubiquitous host–restricted virus with a world-wide seroprevalence between 45 and 100% in adult population [29]. Primary infections of healthy children and adults are frequently asymptomatic but the virus can establish lifelong persistence as a latent infection, from which it can reactivate and spread new infectious particles [30]. Latency is characterized by an absence or low-level presence of virus replication and the appearance of viral genomes as circularized episomes inside the nuclei of bone-marrow cells CD33+ and CD34+ and peripheral blood mononuclear cells [31]. Reactivation of latent forms of the virus, as well as reinfections of the same are common [32], especially for susceptible groups, as immunocompromised patients, pregnant women, newborns, and elderly. Moreover, in some cases there can be sequelae after infection [33].

**Fig. 1:**
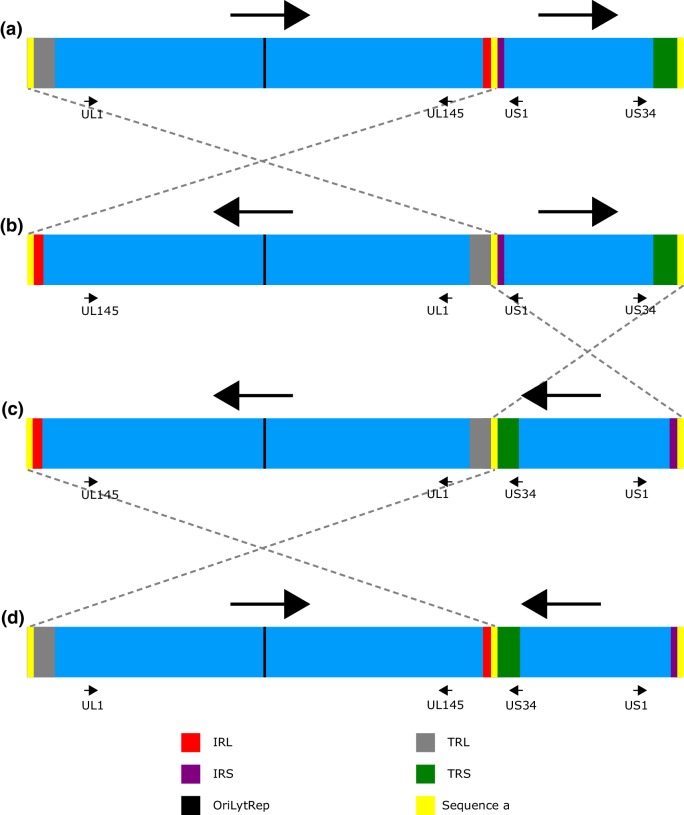
Structure and isomerization of HCMV genome. Representation of the HCMV genome (not on scale) with its four possible isomers (panels **a**–**d**). In panel **a**, the orientation of U_L_ and U_S_ is based on U_L_- and U_S_- orientation of the HCMV *wild-type* reference Merlin (GenBank AY446894). Panels **b**–**d** correspond to the other three possible isomer orientations. Genome regions that are characteristic for HCMV: IR_L_, IR_S_, TR_L_, TR_S_, OriLyt repetition (OriLytRep) and a′ are colored in red, purple, gray, green, black and yellow, respectively. Small black arrows correspond to the direction of selected ORFs (UL1, UL145, US1 and US34) which help to illustrate the orientation of the unique regions (big black arrow) between the different isomers. Dashed gray lines connect the specific a′ sequences that contributed to the isomerization

HCMV consists of a linear double-stranded DNA genome with an average longitude of 235 kbp ± 1.9 kbp (see genome size variation at Table 1), one of the largest of all human-infecting viruses. The GC content of HCMV genome (57.5%) is the highest among human *Betaherpesvirinae*, alike the GC content of *Gammaherpesvirinae* (53.8–59.5%) [34]. The genome is packaged in an icosahedral capsid (*T* = 16) surrounded by a matrix of proteins, the tegument, and enclosed by lipid bilayer, consisting of a mixture between host and virus proteins [35]. Although the genome is linear inside the nucleocapsid, it is circularized during replication; first through *theta*-like replication and subsequently by rolling circle amplification, generating multiple linked copies in tandem [35]. Thereafter, the genome is cleaved, linearized and introduced inside the nucleocapsid, following a *headfull* type packaging [35]. HCMV has a type E genome architecture [36], therefore composed by 2 big inverted domains: long (L) and short (S). In turn, each domain is composed of a central unique region (U, thus U_L_ and U_S_ respectively) and by two repeated regions, one at the terminal end and the other at the intersection with the other unique domain (thus TR_L_/IR_L_ and TR_S_/IR_S_, respectively), resulting in TR_L_–U_L_–IR_L_–IR_S_–U_S_–TR_S_ as a genome organization (Fig. 1). Recombination between repetitive regions is possible, changing the orientation of each unique domain, yielding four possible combinations, thereafter referred as genomic isomers [37, 38] (detailed in Fig. 1). All four genomic isomers can be found in any infective viral population in equimolar proportion [38].

In this review, an overview of HCMV next-generation sequencing (NGS) applications will be given, emphasizing the advances in genomic diversity, strain genotyping, full-length genome methodologies, and coding potential based on transcriptomic and translatomic analysis. In this review, we present the current state-of-the-art and promote future steps in the field.

## HCMV variability

I am very concerned about the use of the same strains, such as Davis or AD169, in different countries and over long periods of time. I wonder how much these could have changed since their initial isolation.—T.H. Weller [39].

HCMV has been regarded as being highly variable between isolates. As early as 1960, T.H. Weller already stated that serological differences between cytomegalic inclusion disease (CID) isolates are sufficiently different to differentiate *classes*, thus being an antigenically heterogeneous group [5]. Later in 1976, Huang and colleagues quantified this variability using DNA–DNA hybridization of 12 different HCMV strains and herpes simplex 1 (HSV-1) and herpes simplex 2 (HSV-2) [40]. It was found that the similarity at nucleotide level was of at least 80%, when comparing different strains of HCMV, in comparison with the 50% when compared to either HSV-1 or HSV-2 [40]. Restriction endonuclease typing also supported moderate divergence between different clinical isolates, without any clear grouping or subtyping between isolates, diverging in concurrence of restriction sites, position and size of the digested fragments [37, 40]. In 1980, Pritchett and colleagues found similar results comparing HCMV AD169 and Towne strains by DNA–DNA hybridization and restriction profiling, implying a similarity of at least 90% at nucleotide level [41].

**Fig. 2:**
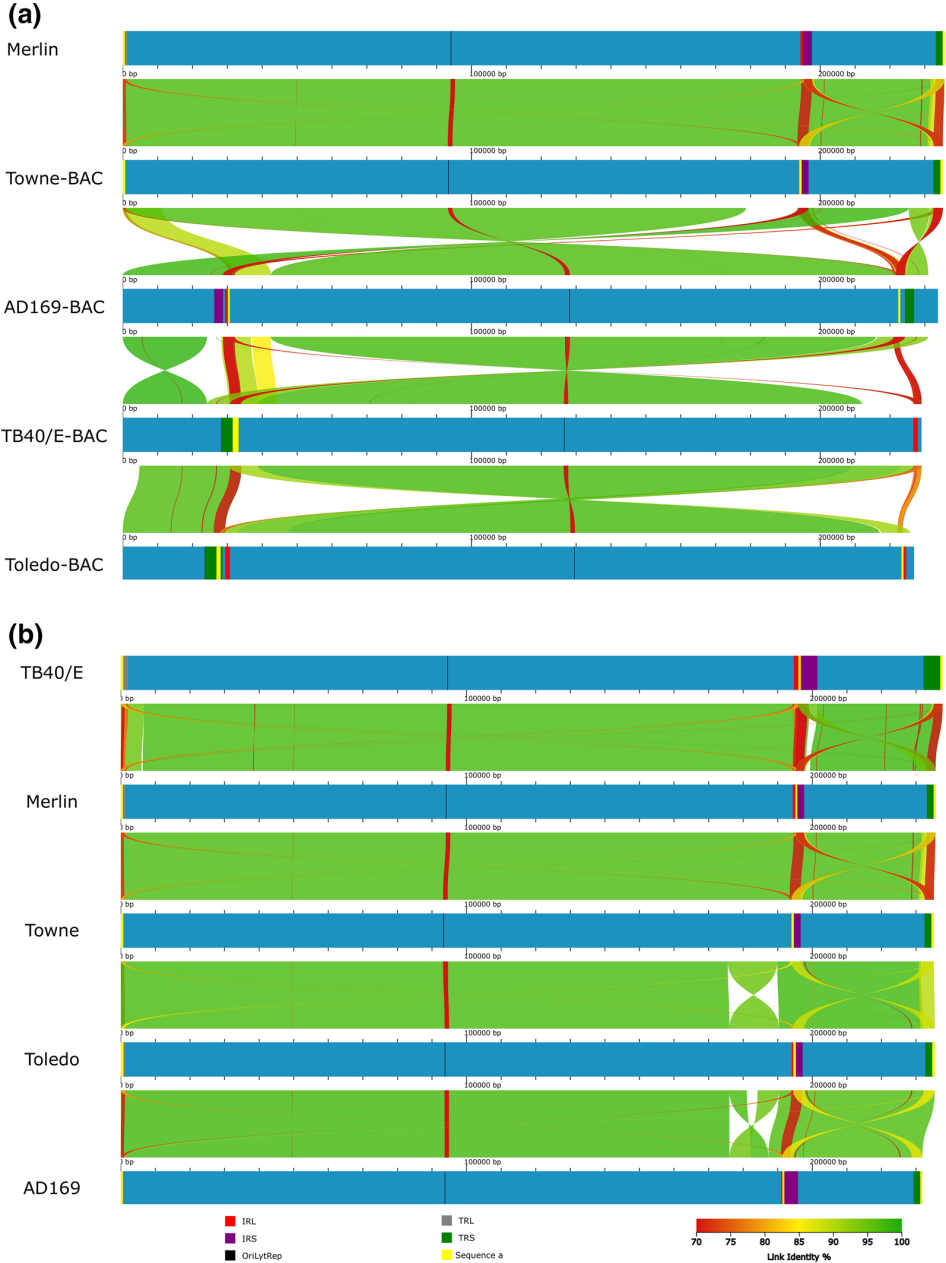
Genome structure of classic HCMV strains. Whole-genome alignments of classical HCMV strains (AD169, Merlin, TB40/E, Toledo and Towne) are presented. Linear maps were build using AliTV visualization software [45], based on whole-genome alignments with Lastz aligner [46]. Both panels **a, b** depict pair-wise comparisons, expressed as percentage of similarity (green to red), that connect different homologous genomic regions. Genomes are pictured in blue. As shown in the legend, the different type E genome repetitive regions (IR_L_, IR_S_, TR_L_, TR_S_, OriLyt and a′ sequence) are colored. Color pattern is shared with Fig. 1 for comparison purposes. Genome length and repetitive regions are on scale. Length units are expressed in base pairs, as shown in the superior part of both panels. Genomes are ordered by descending genome length. Panel **a** represents the pair-wise genome comparison of AD169, Merlin, TB40/E, Toledo and Towne genomes sequenced from BACs (excluding *wild-type* reference Merlin). GenBank accession numbers, ordered as represented in panel **a**, are AY446894, FJ616285, AC146999, EF999921, and AC146905. Panel **b** illustrates the pair-wise comparison of the same strains from panel **a**, but sequenced using NGS short-read technology (with the exception of Towne). GenBank accession numbers, ordered as represented in panel **b** are F297339, AY446894, FJ616285, GU937742, and FJ527563

In 1990, when first feasible applications of sequencing came available, Chee and colleagues published the first version of HCMV genome (AD169 strain, GenBank X17403, Table 1 for related information) [7], which lead to sequence and genome-wide comparison of different isolates and its coding potential [7, 9, 42]. Based on comparative genomics and open-reading frame (ORF) analyses, Cha and colleagues in 1996 discovered 19 genes that were missing from high-passage isolates (strains AD169 and Towne) compared to the low-passage Toledo strain and five clinical isolates. As depicted in Fig. 2, large genomic re-arrangements between AD169 (GenBank AC146999), Towne (GenBank FJ616285) and Toledo (GenBank AC146905) bacterial artificial chromosomes (BACs) can be observed. These re-arrangements, excluding the different possible genome isomers fixed into BACs, are inversions and deletions at the internal end of the U_L_ region, known as the U_L_/b′ sequence, and correspond to missing genes ranging from UL133 to UL154, where several HCMV specific glycoproteins are found in clinical isolates [42, 43]. Likely, U_L_/b′ is lost by recombination and excision with the terminal a′ sequence during long-term passage of clinical isolates, thus changing the levels of virulence and cell tropism of the viral population [42]. AD169 and Towne attenuation is thought to have appeared, partially, as consequence of U_L_/b′ deletion [43]. Later works by Hahn et al. and Bradley et al. described heterogeneous populations of both Towne and AD169 in regards to U_L_/b′ deletion, as well as other mutations [13, 44]. Hahn et al. provided a method for cloning both the Towne varS (GenBank AC146851) and varL (GenBank FJ616285), short and long Towne variants, into BACs as a mean to produce *genetically stable* viral stocks. Towne varS, as AD169, lacks the U_L_/b′ region, meanwhile Towne varL contains U_L_/b′, resembling an uninverted U_L_/b′ sequence from Toledo and clinical isolates obtained in that period [42, 44]. A similar phenomenon is also observed in AD169, one of the most extensively passaged HCMV isolates [13]. In Bradley et al. three AD169 stocks were sequenced: AD169 varUK (GenBank BK000394), AD169 varATCC VR-997 (GenBank AC146999), both derived from NIH 76559 original stock, and AD169 varUC (GenBank FJ527563), using for the first time in HCMV genomics an Illumina sequencing platform. AD169 varATCC proved to be a mixture population of two variants: varS and varL, the later containing U_L_/b′ region, as AD169 varUC. In 2004, Dolan and colleagues sequenced using Sanger method what would become the reference genome for the *wild-type* HCMV, the highly productive Merlin strain (GenBank AY446894), isolated from urine of a congenital infected infant and passaged three times on human foreskin fibroblasts (HFFs) [14]. In addition, Dolan et al. expanded the comparison between isolates with different passage histories, complementing Cha et al. results [42], by defining the genomic features of a *wild-type* HCMV, as opposed to high-passage *attenuated* HCMV strains. The Merlin strain has been extensively used as *wild-type* HCMV reference genome, especially as a backbone for genome annotation and annotation transfer. Since the publication of the first HCMV genome and its coding potential, heterogeneity has been studied either through the genotyping of a selected list of genes, viral markers, or through whole-genome comparisons.

## Genotyping of viral markers

HCMV co-evolved with its human host since diverging from other *Betaherpesvirinae*, circa 120 million years ago [47], and displays a wide array of molecular strategies that allow for survival and perpetuity. All members of the family *Herpesviridae*, but especially HCMV, have acquired functions that favor persistency, immune evasion and molecular mimicry. Some of those functions have been co-opted from host pre-existing machinery [43], as well as other viruses [11, 48] which may account for their considerable genome size. Genes that are linked to persistency, evasion, resistance, or mimicry have been recurrently genotyped in different populations, to assess HCMV variability and its potential thread. These genes of interest, also known as viral markers, can be classified between (i) drug-resistance genes, (ii) virulence, immune evasion, molecular mimicry, and (iii) surface glycoprotein receptors.

Genotyping of HCMV can be distinguished in two approaches: (i) non-PCR and (ii) PCR-based methods. Non-PCR-based methods group direct restriction enzyme digestion [37, 40] and southern blot [41, 42]. Both methods were mostly used in the early days to analyze HCMV variability and to generate the first genetic maps [49]. PCR-based methods group (i) amplicon sequencing and (ii) molecular amplification. Amplicon sequencing has preferentially been conducted with Sanger/dye terminator chemistry sequencing [50–56], whereas variability assessments have been performed using NGS, concretely with second-generation 454 pyrosequencing [55, 57, 58]. Molecular amplification groups PCR techniques that (i) qualitatively and (ii) quantitatively characterize mutations. Qualitative genotyping was predominantly conducted by RFLPs [59, 60]. Quantitative or semi-quantitative genotyping has been exclusively conducted by qPCR [61, 62].

Methods based on restriction enzymes (enzyme digestion, Southern blot, or RFLPs) can fail to detect sequence variability, as only sites sensible to restriction enzymes are analyzed. Conversely, PCR-based methods (including amplicon sequencing) are less prone to miss sequence variability, although only variability found in the amplified region can be studied and poor primer design may reduce the sensibility to detect new variants. Amplicon sequencing has *preferentially* been conducted with Sanger sequencing, as sequencing base accuracy can reach a maximum of 99.999% with this technique [63]. NGS, specifically second-generation pyrosequencing, has also been used for genotype exploration [55, 57]. Despite having a lower base accuracy and read length, it can provide more reads, hence more sequencing depth of the sample to call for multiple variants. Under this scenario, NGS platforms are more informative, due to the higher read yield and their increased sensibility to multiple variants. Genotyping of multiple loci from clinical isolates can be scalable by using amplicon NGS. These sequencing platforms can analyze and later reconstruct different sequences, while keeping traceability of sequence origin by using molecular identifiers, or barcodes. Complete gene genotyping should be considered, as genotyping only specific regions of the gene increases the likelihood to lose unknown polymorphic sites [64] or to overlook new recombining genotypes between different polymorphic sites, as already been described for UL55 (gB) [65]. Other existing sequencing platforms have yet to be tested on HCMV amplicon genotyping, as sequencing technologies improved fast and full-length genomes where soon available.

Currently, there is no consensus on the classification of HCMV strains based on genotype, evolutionary relationship, or clinical relevance. Loci genotyping should proceed with caution, as the costs of sequencing a full-length HCMV genome have decreased in the last years. Not aiming to sequence a full clinical isolate genome might be an unrepairable opportunity to understand this complex pathogen. Whole-genome sequencing can simultaneously capture all variants and remove the need to design and optimize PCR assays for multiple variant detection, allowing e.g., for a parallel antiviral-resistance testing in a single experiment [66] or for predicting changes to epitopes for vaccine development [67].

## Next-generation sequencing in HCMV research

…[A] knowledge of sequences could contribute much to our understanding of living matter—F. Sanger [68].

Since the apparition of the first massively parallel sequencing technologies in the 2000s, new possibilities for HCMV research emerged after each technological breakthrough. 454 Life Sciences, later known as Roche 454, and Illumina Inc independently created the first massively parallel sequencing platforms, used in the first deep sequencing on HCMV [13]. These technologies, not only created a new way to recompose full-length HCMV genomes without sequence cloning, but allowed a better understanding of its population variation and coding capacity during infection [69]. Recently, a new opportunity to differently understand the HCMV genome has appeared with the application of third-generation sequencing, based on long-read real-time sequencing [27, 70, 71].

### Whole-genome sequencing

Up to the submission of this review, 305 full-length *distinct* HCMV genomes have been published (NIAID Virus Pathogen Database and Analysis Resource, ViPR) [8], 251 of them derived from clinical isolates (GenBank accession numbers and sequence relevant information can be consulted at Table 1), and of these sequences only 205 correspond to unpassaged or low-passage isolates (< 4 passages). Since Chee et al. published the first HCMV genome, Sanger sequencing has been regarded as the standard for HCMV drug-resistance detection [72].

Currently, the most precise full-genome cloning system consists of an embedded complete genome in a BAC with Cre/LoxP self-excising system, amplifying the genome in a bacterial system with very low mutation rates, as the BAC is amplified by the bacterial DNA polymerase. Cre/LoxP self-excising system does not modify the original virus sequence with the exception of a 34 bp insertion downstream of the US28 gene [73]. Although, BAC cloning can produce long-lasting stable strain amplification systems [20, 73], cloning and sequencing by *primer walking* can be time-consuming, inefficient and might not be an optimal method for exploring virus diversity within a clinical sample. Interestingly, HCMV genome BAC cloning captures genomes individually, as they are contained inside the viral particles, creating *fixed genetically stable* viral genomes. These stable genomes faithfully represent the individual variants of that viral particle, including its genome isomerization (represented in Fig. 1), as well as multiple genomic variants, which may not necessarily represent the most abundant form in the viral population or its infective capacity. In Fig. 2, many genomic re-organizations become apparent when comparing different HCMV strain genomes. In Fig. 2a, different BAC isolated HCMVs are represented, characterizing inversions spanning the entirety of unique regions when comparing two genomes. These apparent inversions are in fact a result of comparing different HCMV isomered genome sequences, fixed and stabilized in BACs (i.e. TB40/E—BAC vs. Toledo—BAC, in which U_L_ has different directions). In addition, in Fig. 2a, inversions or translocations that reorganize the classical structure TR_L_–U_L_–IR_L_–IR_S_–U_S_–TR_S_ should be taken with caution, as they may arise from the introduction of the genome into the BAC vector. AD169-BAC (GenBank AC146999) offers a clear example, as its U_S_ region appears to be fragmented and translocated to the terminus of the genome. Once these previous re-organizations are considered, other re-arrangements can be recognized in Fig. 2. These re-organizations arise from imperfect homologous recombination during HCMV genome replication, being focus of HCMV infective variation studies. Interestingly, these re-organizations can be found in both Fig. 2a, b, as the same strains are illustrated in both panels but with their genome sequence is derived from either BAC cloning and posterior sequencing or by second-generation sequencing from a pool of viral particles. This comparison between both sequencing methods exemplifies the differences between re-organizations derived from (i) technical procedures (BAC cloning), (ii) viral replication (genome isomers), or by (iii) imperfect homologous recombination and mutation. In this regard, the deletion (and/or inversion) at the U_L_/b′ region, characteristic for high-passage *attenuated* strains, can be observed when comparing AD169, Towne and Toledo strains in both panels of Fig. 2, as previously discussed in this review.

Despite the benefit of capturing, fixing and genetically stabilize single viral genomes that BAC cloning can offer, most of the partial or full-length genomes have been derived from second-generation sequencing platforms, mainly due to BACs poor scalability for viral population research. These platforms enabled the discovery of different variants in HCMV viral populations (as previously discussed for Towne varS and varL) [13], and a substantial decrease in time and resources needed for genome sequencing. High-throughput NGS allowed to increase the number of clinical HCMV genomes to more than 170. Sequencing of full-length HCMV genomes was initially performed with Roche 454 pyrosequencing [17, 19, 25], coupled to either or both Sanger and Illumina sequencing to polish low quality regions, producing 57 HCMV genomes. Illumina sequencing platform rapidly outperformed its competitors with its improved chemistry, yield and base quality, generating most of the available genomes (158 out of 251) [15, 20, 22–24, 26]. Albeit NGS boosted the sequencing of HCMV genomes, direct sequencing of clinical HCMV remained an issue, due to its low viral particle yield of during infection. Common sources of clinical material for HCMV sequencing include: blood, urine, bronchoalveolar liquid, tissue (mostly kidney or liver), and amniotic fluid (a relationship between sequenced genomes and tissue of isolation can be found in Table 1).

Short-read second-generation sequencing provides a solid working approach to the study of single-nucleotide variants (SNVs) due to its high read yield, increased read coverage along the genome and improved sequence error (correction, improving variant detection). Unfortunately, the characterization of genome re-arrangements or structural variants (SVs) with second-generation sequencing can be challenging due to (i) its association with (low-complexity) repetitive regions, (ii) the difficulty of short-reads to span large genomic events, and (iii) to *precisely* localize breakpoint coordinates [74].

The reconstruction or assembly of a HCMV genome can be a complex task as (i) clinical material has low viral genome copy numbers, directly affecting sequencing coverage and the overall genome quality. Additionally, (ii) HCMV genome contains three regions with low-complexity repetitions at the unique terminal and unique internal end, increasing the difficulty to *correctly* align and recruit reads during genome assembly. Finally, (iii) mixed HCMV populations are expected, either as a result of co-infection of different strains or activation of latent HCMV infections, generating a genetically heterogeneous (or heteroclonal) population [55]. Discerning which variants co-concur (co-linearize) and belong to the same viral genome may benefit the examination of clonal heterogeneity of the viral population.

Different techniques have been coupled to second-generation sequencing platforms, to increase the yield of viral reads. Most strategies use (i) multiple sequence displacement amplification (MDA) [19, 22] to increase the input viral DNA in the sample, or (ii) target enrichment to enrich the sample by capturing viral DNA using DNA or RNA probes (also known as *bait libraries*) [26, 66, 70, 75]. MDA kits use high-fidelity polymerases (generally a ф29 polymerase) in conjunction with a set of random hexamers to amplify DNA at isothermal conditions [19, 76, 77]. Although, this technique amplifies viral genomic fragments between one to three orders of magnitude [19], biases have been reported specifically linked to a high allelic drop-out effect (ADO, preferential amplification of a subset of alleles in a heteroallelic sample) and non-uniform amplification of linear double-stranded DNA (related to the GC content of the amplified sequence) [19, 76, 78]. Both Marine et al. and Roux et al. conducted genome coverage analysis on MDA dsDNA amplified viruses [76, 78], providing clear evidence that MDA amplification is one of the disturbing factors in completing a full-length genome. A recent study by Borgström and colleagues compared four different MDA available kits during single-cell human DNA amplification: AMPLI1, MALBAC, Repli-G and PicoPlex, taking coverage, SNP calling and ADO to test the reliability of the kits [77]. Borgström et al. showed that Repli-G produced the most uneven low coverage genome amplification, followed by PicoPlex. AMPLI1 and MALBAC had comparable even coverages [77]. SNP calling performed poorly by Repli-G kit, only 3% of the variants were detected, in comparison with the 25% detected by MALBAC [77]. The Repli-G ADO effect is probably linked to the poor performance during SNP calling. Only one allele in all studied loci and replicates was detected [77]. Target enrichment, conducted mainly with SureSelect^XT^ library enrichment, has been used to obtain over 50 unpassaged HCMV genomes [26, 66, 70]. By designing custom bait libraries that cover the entire HCMV genome, it is possible to capture (by hybridization and streptavidin bead separation) the fraction of a given NGS library that corresponds to the virus, and further amplify it [79]. This technique allows to sequence viral genomes *directly* from clinical samples, avoiding virus culturing (used to increase the yield of the virus at cost of virus adaptation to the growing cell line [16]). SureSelect^XT^ enrichment has been extensively used in the last years [23, 26, 66, 70, 80]. There are at least two different custom bait libraries being currently used, one developed at the Center for Virus Research, University of Glasgow [26] and a second designed by the PATHSEEK consortium, jointly with Oxford Gene Technology™ [23, 66, 70], albeit none of them is publicly available. Both MDA and target enrichment rely on additional PCR amplifications, hence susceptible to introduce a new sequence bias to the sequencing library. Regardless of the increased HCMV sequencing performance that both techniques offer, the omission of infrequent viral variants should be a cause of concern. MDA methods, especially Repli-G, have a known preference to amplify certain regions and variants, leading to uneven low coverage regions and narrowed variant diversity, hence likely over-looking the intrinsic variation in a viral population. Theoretically, a narrowed variant diversity could also be found if target enrichment was used, as its efficiency relies on a library design for *known* but also *unknown* variants.

Assembly of herpesviruses, such as HCMV, can be inaccurate due to its low-complexity repetitive regions as well as its local deviant GC content, producing discontinuities, or gaps, in the assembly [15]. This inaccuracy is linked to the read length [74]. A longer read size is more likely to produce reads that can span or bridge regions where the library or sequencing platform might be infra-represented, and correctly characterize repetitious regions, both its boundaries and number of repeats [74].

Poor connectivity between *distinct* assembled regions, or contigs, is a major challenge for assembling full-length HCMV genomes. As previously stated, assembly inaccuracy can be linked to low-complexity repetitive regions as well as a local deviant GC content [74, 81], such is the case for type E genomes like HCMV. GC deviant regions may produce read miss-representation in those regions, potentially failing to assemble a full-length genome. Different parts of the sequencing scheme can be responsible for this phenomenon, PCR amplification of the library, cluster amplification, or the reading during sequencing [81]. However, library amplification by PCR plays the major role in generating GC bias, especially affecting short-read synthesis-based sequencing (i.e., Illumina sequencing platform) [81]. Likewise, low-complexity repetitive regions can impair an assembly by generating multiple possible positions where reads could align and in that way, generating new sub-alignments, increasing the complexity of the assembly and reducing the accurateness of certain regions [74]. Poor region connectivity (i.e., an assembly with too many short contigs) can challenge full-length HCMV genome assembly when different viral variants are present in the same sample. Improving this connectivity would increase the recovery of *complete* and *distinct* genomes at sub-strain level, as well as sequencing through repetitive regions [74]. Consequently, longer reads are desirable, as the longer a given read is, the longer the contigs in the assembly would be, hence increasing the assembly connectivity. Furthermore, improved read-length would likely increase the chance to contain multiple (*distant*) variants in the same read, providing direct evidence of their co-linearity in a given single virus genome from a clonal heterogeneous HCMV population.

Third-generation long-read sequencing platforms, such as SMRT™ from Pacific Biosciences™ and Nanopore sequencing from Oxford Nanopore Technologies™ open the possibility to improve assembly connectivity, providing a promising platform for single virus (partial) genome sequencing, due to its extended read-length. Until the submission of this review, only 3 different long-read HCMV sequencing projects have been published, 2 in 2017: (i) Balázs and colleagues with a hybrid approach using the Pacific Biosciences™, PacBio RS II system and Oxford Nanopore Technologies™, MinION™ platform [27, 28, 82], and (ii) Eckert et al. with the Oxford Nanopore Technologies™, MinION™ platform [70]. The last project, published in 2018, by Karamitros and colleagues, also used Oxford Nanopore Technologies™, MinION™ platform [71]. In Balázs et al., HCMV Towne varS strain (passaged more than 125 times with the 180,887–191,406 region of the original genome substituted with the 1–11,996 region, GenBank LT907985) full-length genome was assembled using cDNA transcripts and the reference Towne (GenBank FJ616285) [27, 28]. In Eckert et al., HCMV genomes were sequenced, *directly* from clinical material, with and without target enrichment (SureSelect^XT^, without downstream amplification). In contraposition with Balázs et al., only 1.2% of the reads (from non-enriched samples) could be assigned to HCMV; meanwhile for viral enriched samples, 98.7% of the reads were assigned to the virus, reconstructing the HCMV genome up to 99.4% with a mean coverage of 89.9× [70]. In Karamitros et al., HCMV TB40/E strain (GenBank EF999921) clone Nano, was sequenced using cell-associated replication, without in vitro amplification, and a posterior viral concentration with ultracentrifugation. A 48-h run produced close to 47,000 reads with a uniform average coverage of 100×. The full-length genome was obtained by a co-assembly (or “hybrid de novo assembly”, as the authors refer to) of the same filtered reads by a fast long-read assembler, SMARTdenovo [83], and a short-read assembler, SPAdes [84]. With this approach Karamitros et al. (raw MinION™ data are available at NCBI—SRA project number PRJEB25285) provide for the first time, using NGS, evidence of viral genome isomers from a type E genome [71]. Long-read sequencing enabled Karamitros and colleagues to find several SVs in a BAC-derived TB40/E polyclonal sample: (i) a deletion spanning UL144–UL145, (ii) one inversion, (iii) two relocations, and (iii) several short insertions or deletions (deriving in local misalignments) [71].

Hitherto, no method combines all characteristics to analyze variation in a HCMV polyclonal infection as, or close to, single virus genomes. The ideal method for studying HCMV would have to (i) sequence *directly* from clinical material (no cell or in vitro amplification), (ii) to be *unbiased* (either by enrichment or uneven amplification), and (iii) to provide direct evidence of variant *co-linearity* to an individual viral genome.

### Transcription, translation and regulation analysis through RNA-sequencing

Since the publication of the first studies of HCMV transcriptomics by Gatherer and colleagues, the advancements on HCMV RNA-sequencing have highlighted new aspects on its behavior: regulatory small RNAs [85, 86], new RNA splice variants [27, 69] and newly detected ORFs [69, 87]. Early estimates ranged from 164 ORFs [14, 69, 88] to 220 ORFs [89], although ribosome profiling identified up to 751 individual ORFs [87]. Those 751 translationally active ORFs may be a more precise estimate of coding capacity, as it is likely to account for over the (i) 100 splice junctions that HCMV genome contains [69, 87], (ii) transcript polycistrony (i.e., UL138) [90] and (iii) short ORFs [87]. Despite the obvious codifying complexity of HCMV, the *wild-type* reference Merlin (GenBank NC_006273.2) currently has 173 annotated genes, of which 168 are protein-coding genes and 5 non-protein-coding genes.

According to Gatherer et al. (BioProject PRJEB2543, see Table 2 for additional project information) 3 different types of transcripts can be expected when analyzing HCMV infections: (i) protein-coding transcripts, (ii) non-coding non-overlapping transcripts (RNA2.7, RNA5.0, and RNA1.2 long non-coding RNA or lncRNA), and (iii) antisense transcripts (transcribed antisense with respect to protein-coding regions) [69, 85]. Studying the infectious behavior of HCMV Merlin strain in HFFs at 72 h post infection showed that the presence of antisense transcripts throughout the HCMV genome, by strand-specific RNA-seq (in [69] referred as “directional sequencing or DDS”) and strand-unspecific RNA-seq (in [69] referred as “non-directional transcript sequencing NDS”), represented a 8.7% of the overall transcription [69]. In addition, RNA2.7, RNA5.0, and RNA1.2 transcripts account for 65.1% of the overall transcription, especially RNA2.7 that represents the 46.8% of the overall transcription. Strikingly, protein-coding transcripts only account for a third of the overall transcript production [69]. New splicing sites were discovered, leading to the description of new alternative splicing events and confirmation of four novel gene transcripts (RL8A, RL9A, UL150, and US33A) [69]. A year later, Stern-Ginossar and colleagues (BioProject PRJNA177721) used ribosome profiling (sequencing of mRNA protected within the ribosomes) to study Merlin transcription in HFFs at 5, 24, and 72 h post infection [87]. 751 translated ORFs were found with only 147 being previously described. These novel putative ORFs where derived from (i) nested ORFs (in and out of frame), (ii) upstream short ORFs, (iii) antisense ORFs, and (iv) short unpredicted ORFs (ORFs coding for protein between 100 and 200 aminoacids [91]). Multiple ORFs were translated from RNA1.2, RNA2.7 and RNA4.9 lncRNA, acting as a precursor polycistronic mRNA [87].

**Table 2:** HCMV RNA-sequencing projects

BioProject number	Study name	HCMV strain	Host cell line	Temporal data	Sequencing platform	Library source	Sample number	References
PRJNA421010	miRNA-mediated targeting of human cytomegalovirus reveals biological host and viral targets of IE2	TB40/E	MCR-5 and THP-1	1–10 days	Illumina	cDNA	16	[92]
PRJEB25680	Dual-platform long-read RNA-sequencing of the human cytomegalovirus lytic transcriptome	Towne varS	MCR-5	1, 3, 6, 12, 24, 72, 96 and 120 h	Oxford Nanopore Technologies	cDNA	12	[82]
PRJEB22072	Transcriptome analysis of the human cytomegalovirus using pacific biosciences RSII platform	Towne varS	MCR-5	1, 3, 6, 12, 24, 72, 96 and 120 h	Pacific Biosciences	cDNA, oligo-dT, random PCR	26	[27, 28]
PRJNA389726	Transcriptome-wide characterization of human cytomegalovirus in natural infection and experimental latency	TB40/E, ∆*UL135*, ∆*UL138* and 12 latent clinical isolates	CD34+, PBMC	2 and 6 days	Illumina	cDNA	39	[93]
PRJNA388483	Cellular responses to human cytomegalovirus infection	TB40/E	MRC-5 and ARPE-19	24, 72 and 120 h	Illumina	cDNA	12	[94]
PRJNA373848	Transcriptome analysis of HCMV infected tissues	TB40/E	ARPE-19 and decidual tissue	1 or 7 days	Illumina	cDNA	4	[95]
PRJEB15199	HCMV transcriptome in primary monocyte-derived cell types	TB40/E	PBMC	72 h	Illumina	Oligo-dT	33	[96]
PRJNA342503	RNA binding protein CPEB1 remodels host and viral RNA landscapes [RNA-Seq]	TB40/E, Towne	HUES9, H9 and HFFs	48 and 96 h	Illumina	cDNA	6	[97]
PRJNA304028	microRNA expression analysis of CMV infected human fibroblasts in two cultures	AD169	HELF-977 and HAF-1608	0 and 3 h	Illumina	cDNA, size fractionation	2	[98]
PRJNA299678	Transcriptome analysis of diverse cell types infected with human cytomegalovirus [RNA-Seq]	TB40E and Towne	HFF, EC and NPC	2 and 8 h	Illumina	cDNA	22	[97]
PRJNA269099	MicroRNA targetome analysis during HCMV infection	Towne varL	HFF	0, 24, 48 and 72 h	Illumina	cDNA, size fractionation	40	[99]
PRJNA177721	Decoding human cytomegalovirus using ribosome profiling	Merlin	HFF	5, 24 and 72 h	Illumina	cDNA	16	[87]
PRJNA148583	High-resolution profiling and analysis of viral and host small RNAs during human cytomegalovirus infection	Towne	HFF	24 and 72 h	Illumina	cDNA, size fractionation	2	[85]
PRJEB2543	High-resolution human cytomegalovirus transcriptome	Merlin	HFF	24 h	Illumina	cDNA	2	[69]
PRJNA340198	Gene expression of human THP-1 cells infected by cytomegalovirus	Towne	THP-1	4 days	Sanger	cDNA	3	[100]
PRJNA257463	Ribosome profiling reveals pervasive translation outside of annotated protein-coding genes	Unknown	Unknown	Unknown	Illumina	cDNA	4	[101]
PRJNA394123	Defining the transcriptional landscape during cytomegalovirus latency with single-cell RNA-sequencing	TB40E	HPC	3, 4, 5, 6, 7 and 14 h	Illumina	cDNA	7	[102]

Relation of all available HCMV expression and translation projects using RNA-seq data extracted from (NCBI, July 2018). Relevant information is attributed to each experiment by its BioProject number, which can be used as unique entry point to access the raw data produced in the RNA-seq experiment. Each RNA-seq experiment is linked to the type of library, sequencing platform, number of samples run in the experiment, as well as the name and basic conditions of study (HCMV strain, cell line and time of infection that was analyzed)

MicroRNAs (miRNAs) are small RNAs of 22 nucleotides long, transcribed by RNA polymerase II [92], related to RNA silencing and post-transcriptional regulation of gene expression. Both functions have been studied for their possible regulatory role during an HCMV infection [10, 85, 103]. While miRNAs are known to be non-immunogenic, some are known to have a regulatory function in viruses [86]. HCMV is known to produced mature miRNAs during infection [10, 103]. Stark and colleagues (BioProject PRJNA148583) studied host (HFFs) and HCMV Towne miRNAs profiles at 24 and 72 h post infection. Up to 20% of the miRNAs were from viral origin, providing evidence of 22 miRNAs being incorporated into the endogenous host silencing machinery [85]. In contrast, Meshesha and colleagues described the fraction of miRNA dropped to only 5% [86]. Even if Stark et al. and Meshesha et al. identified the same seven top most abundant transcripts (miR US5-2-3p, US25-1-5p, US25-2-3p, US25-2-5p, UL22A-3p, UL22A-5p, and UL36-5p), their abundances substantially differed. Those changes in abundance could be attributed to 3 different causes: firstly, (i) two different HCMV strains were used (Towne in Stark et al. vs. AD169 and 3 clinical isolates in Meshesha et al.). Secondly, (ii*)* RNA was collected at different time points (72 h vs. 96 h post infection), and finally (iii) different methods of miRNA assignment were used (mapping reads with Bowtie v0.12 to miRBase v16.0 vs. mapping reads with BWA v0.5 miRBase v17.0) [85, 86]. Using previously published ribosome profiling data by Stern-Ginossar et al., Ingolia and colleagues (BioProject PRJNA257463) found evidence of novel polypeptide production in RNA2.7 transcript, capable to induce immune responses from the host [101]. Kim et al. (BioProject PRJNA269099) found that a large fraction of human miRNAs targets was shared with viral miRNAs in HFFs infected with Towne varL after 24, 48 and 72 h post infection [99]. In 2016, Buzdin and colleagues (BioProject PRJNA304028) could link a complete suppression of host miRNAs regulation during early stages (3 h) of an HCMV infection, by infecting embryonic lung fibroblasts (HELF-977) and skin fibroblasts (HAF-1608) with AD169 [98]. Lastly, Stark et al. found evidence of miRNAs being derived from the lncRNA RNA2.7, contributing to profile HCMV long RNAs as precursors to other functional RNAs. Interestingly, Stern-Ginossar et al. found similar results applying ribosome profiling techniques, describing lncRNAs as precursors for putative short proteins [87].

Transcriptomics can also be used to understand the processes of cell tropism and infection in different cell types. Van Damme et al. (BioProject PRJEB15199) compared differences in expression between TB40/E infected macrophages and dendritic cells (DCs) derived from whole blood donations [96]. Interestingly, in primary cell types, differentially expressed genes often belong to clusters, suggesting a functional coordination between those transcripts coming from genes of the same family. Concretely, the decrease in expression of RL11–RL13–RL14–UL1 and UL4–UL11; and the increase of UL120–UL121, UL148D–UL149, and US33–US34A in DCs were strikingly pronounced [96]. In macrophages type 1, the cluster UL81–UL86 appeared to have its expression generally decreased (although UL81–UL82 and UL85 did not reach clear significance). Contrary, in macrophages type 2, the same cluster, UL82–UL86 had its expression increased (UL84 was not significant), as well as RL11–RL12, UL2-3, and UL148A–UL149 loci. Similarly, the unique short region US7–US9 had their expression increased [96]. Possibly, US1–US6 region would have its expression increased, as the whole region is generally related to immunomodulation, but was deleted in the production of TB40/E BAC on which Van Damme and colleagues based their study. As expected, most of the differentially expressed genes were related to immunomodulation, cell tropism (prominently UL74, US9, and US27) and adaptability to different cell types (as UL4 and UL5) [96].

Batra et al. (BioProject PRJNA342503) proposed some advances on alternative therapeutic targets in 2016 [97]. Cytoplasmic polyadenylation element binding protein 1 (CPEB1), responsible for cytoplasmic polyadenylation, was found to have a major role in infection-related cytopathology and post-transcriptional changes in different strains of HCMV (TB40/E and Towne) and in Herpes Simplex virus 2 in different tissue types [97]. Decreased transcription levels of CPEB1 reduced viral RNA polyadenylation (shortening poly-A tails), alternative splicing and other RNA processing events, which leaded to a decrease of HCMV titers and shift in the transcription profile in comparison with a mock infection [97].

Although pending for experimental validation, Zhang and colleagues (BioProject PRJNA340198) described the latent HCMV Towne infection cell transcriptome in THP-1 cells [100], defining more than 2000 host differentially expressed genes, with approximately half of them with an upregulated expression profile. As expected, those differentially expressed genes were involved in pathways of apoptosis, inflammatory response and cell cycle progression [100]. Interestingly, lncRNAs were differentially expressed with an ongoing HCMV latent infection [100]. A year later, Cheng et al. (BioProject PRJNA389726) compared the expression of natural infection (healthy peripheral blood mononuclear cells latently infected with clinically uncharacterized HCMV) and experimental latency system in a transcriptome-wide study using positive strand SureSelect^XT^ target enrichment [80]. The experimentally latent system used mutated TB40/E strains: ΔUL135–TB40/E (latent-like) and ΔUL138–TB40/E (strict-lytic). The SureSelect^XT^ enrichment represented a viral RNA increase between 74.35 and 81.2%, increasing viral read yield more than 6000 fold, without biasing the read distribution of the transcriptome [80]. Strikingly, *wild-type* TB40/E and recombinant ΔUL135 were very similar in transcript composition and abundances [80]. Alternatively, recombinant ΔUL138 infected cells harbored transcripts being antagonistically expressed in *wild-type* or ΔUL135 infected cells. Finally, the authors proposed a list of 30 core differentially “low to moderate levels” expressed genes in HCMV latent samples (ΔUL135 or clinical latent samples). Unfortunately, no lncRNA were analyzed [80]. Shnyder and colleagues (BioProject PRJNA394123) used publicly available datasets and single-cell transcriptomics to define HCMV latency dynamics in infected cell populations. Interestingly, Shnyder et al. did not find any “clear restricted latency-associated expression program” [102] or set of genes, that could clearly explain the transitions from lytic-to-latent or latent-to-lytic during infection. Furthermore, transcription levels in latent cells resembled more those of very late infection, with low to medium transcription rates. This overall change in transcriptional rate, as cause of transition between the two states, apparently conflicts with Cheng et al. 30 latency-associated candidate genes list [80]. Further research is needed to understand the dynamics of latency in HCMV.

In 2017, Balázs and colleagues (BioProject PRJEB22072) reported the first HCMV transcriptome sequenced with long-read technology, the SMRT Bell™ Pacific Biosciences™ single molecule consensus platform. In this study, more than 291 novel transcript isoforms, 13 transcriptional starting sites (TSS), 22 transcriptional ending sites (TES) and 11 novel splicing events were characterized [27]. Most isoforms displayed unique combinations of ORFs, modifying the length of the transcript. Most of the length differences between isoforms were caused by an N-terminal truncation, losing an additional ORF upstream of the main ORF. Moreover, 8 novel antisense transcripts to canonical ORFs (UL20, UL36, UL38, UL54, UL115, US1, US17, and US30), and a new partially antisense transcript (RS2) in the short repeat region [27]. Balázs and colleagues also described transcript diversity in UL38 locus (i.e., hypothetical UL38A, longer form of UL38 with a putative non-canonical start codon), which has already been hypothesized to have a role in latency-to-replication transition in Cheng et al. [80]. Oxford Nanopore Technologies™ direct RNA-sequencing could provide extra evidence, as it can allow for (i) sequencing RNA *directly* (no retrotranscription or amplification) and (ii) keeping *strand specificity*. Additionally, (iii) it permits sequence transcripts with very *different fragment sizes* (as opposed to SMRT Bell™ Pacific Biosciences™ approach in Balázs et al. where analyzed transcripts range between 1 and 2 kbp).

## Conclusions and future challenges

Almost 30 years have passed since the first full-length genome of HCMV was published [7], and the amount of knowledge gathered with different NGS experiments has been invaluable to detangle the nature of this ubiquitous virus. Even with all information that has been collected and technologies developed, some challenges are yet to be addressed: (i) the centralization and integration of information, and (ii) the production of improved assemblies, notably in complex clonally heterogeneous samples.

HCMV genome-, expression- and translation information is scattered in literature, but by (i) improving protein orthology, (ii) collecting and unifying clinical data, and (iii) creating a dynamic and collaborative annotation environment, the scattered available information may be reconstructed and contextualized, providing a valuable broad picture of HCMV. Different annotation nomenclatures have existed for the past years [7, 104] and recently, a new protein orthology has been published [8]. This new annotation, promoted by ViPR, is based on Domain-architecture Aware Inference of Orthologs (DAIO, Forester library) [105], and already available phylogenetic classifications, offering a *manually high-quality curated* database of Strict Orthology Groups (SOG). Orthology groups may help to identify and classify new *Herpesviridae* genes and to understand the functional differences between the different orthologs.

Over the years, the number of sequenced clinical isolates has greatly increased, albeit clinical metadata linked to the viral isolates (i.e., gender, age, patient cohort, ethology of the disease or isolation year) has not. Most of this information remains unavailable or heavily scattered in the bibliography. Some resources, as ViPR [8], provide centralized access to part of this metadata by automatically accessing GenBank records, although it remains incomplete as relies on non-standardized GenBank entries. An environment to deposit relevant clinical data with the corresponding viral information (i.e., isolate characteristics and genome) would provide high-quality information, helping to identify pathogenic determinants, as already has occurred for other viruses [106]. Currently, most of HCMV genome and transcriptome are annotated by automatic or semi-automatic tools, based on pre-existing references (custom databases or annotation transfer tools, such as RATT [107]). Unfortunately, not all annotations are updated with the current discoveries in HCMV expression and translation. A centralized and integrative RNA-seq platform would benefit the current state of annotation, as it would offer a constantly updated HCMV annotation contextualizing the available evidence from different experiments.

Finally, assemblies can be improved using different strategies, although *connectivity*, as previously discussed in this review, is one of its key aspects. Long-read technologies cannot only, *connect* scattered or unfinished regions of HCMV assemblies and characterize complete transcription events; but it can also provide a better understanding of structural and point variation in HCMV infections. Recently the term “ultra-long reads” (ULR), reads longer than 100 kbp, has been introduced [108]. Theoretically, ULR could (partially) cover any of the unique regions (U_L_ or U_S_) of HCMV, or in exceptionally cases, bridging both unique regions, as reads longer than 1 Mbp have already been reported [108]. Reads longer than 100 kbp may help to *unambiguously* connect distant variants from a clonally heterogeneous HCMV population.
